# Parliament and the Profession

**Published:** 1917-09-01

**Authors:** 


### Parliament and the Profession.

### Manufacture of Artificial Limbs.

The Minister of Pensions (Mr. Barnes) informed Major Kerr-Smiley that action is at present being taken to obtain details of a new process, known as "Dr. De Page's system for making artificial limbs," which has recently been adopted at the Belgian Hospital at La Panne, and the matter will be considered as to whether it is advisable to apply the process to the manufacture of limbs in this country and to take advantage of the kind offer of Dr. De Page and the Belgian authorities to train disabled soldiers in the process of manufacture.

### Indian Military Station Hospitals.

Mr. Montagu informed Major Davies that the outbreak of war prevented effect being given to the recommendations of a committee under the presidency of Sir P. Lukis which prepared a detailed scheme for the introduction of the station hospital system for Indian troops. It has now been definitely decided to introduce the station hospital system for Indian troops at the earliest possible moment. For British troops in India the system has been in existence for many years.

### Hospital Ships.

The Minister of Blockade (Lord Robert Cecil), in answer to inquiries by Mr. Gilbert and Mr. T. Wilson, stated that the English and French Governments had agreed that hospital ships shall carry a neutral Commissioner appointed by the Spanish Government. It is hoped that this may put an end to enemy attacks, although a definite assurance to this effect has not yet been received from the enemy Governments, to whom, as required by the Hague Convention, the names of British hospital ships have been communicated, and the same is, no doubt, the case as regards hospital ships of Allied Powers.

### Irish Dispensary Medical Officers.

Mr. Muldoon inquired of the Chief Secretary for Ireland what steps are taken by the authorities when it is found that dispensary medical officers in Ireland are unfit, for health reasons, efficiently to discharge their duties. He was informed by Mr. Duke that these officers are visited annually, and their work inspected by the medical inspectors of the Local Government Board. Should a doctor develop any ailment, such as deafness, to an extent to render him unfit to perform his duties, the fact would come quickly to the knowledge of the Guardians, who would report to the Board, and on it being shown to their satisfaction that the doctor had become unfit for office he would be required to resign. The Guardians are empowered to grant superannuation allowances in such cases.

### Pay of Naval Surgeons.

Dr. Macnamara informed Mr. Hohler that it has now been decided by the Admiralty that no deductions shall in future be made from the pay of naval surgeons who had retired before the war with a gratuity and had placed their names on the emergency list, and that deductions already made from the pay of such officers shall be refunded.

### Typhoid Outbreak in Carmarthenshire.

Mr. Hayes Fisher informed Major Davies that a medical inspector of the Local Government Board, who had visited the Tamble district, Carmarthenshire, to inquire into the recent outbreak of typhoid, had reported that it was caused by infected milk, and had advised the local council as to the action to be taken to prevent the disease recurring.

### Unregistered Dentists.

Mr. Raffan asked the Prime Minister whether he had received a memorial signed by nearly 200 members of Parliament asking that the Departmental Committee on Unregistered Practice in Dentistry should be reconstituted to include representatives of unregistered practitioners, and also a larger representation of members of the House of Commons; and, if so, whether it is proposed to give the additional representation asked for? Mr. Bonar Law answered that the petition had been received, and it is hoped that it may be possible to take action in the direction suggested by Mr. Raffan.

### Rising of Parliament.

Parliament rose on August 21 until October 16. The session will continue until Christmas.

### A Collection of Officers' Portraits.

The Committee of the National War Museum asks for unmounted copies of photographs of officers serving, or who have served, in the present war, accompanied by concise biographical notes as to dates of promotion, distinctions, etc.

